# Value of MRI T2 FLAIR Vascular Hyperintensities Combined with DWI ASPECTS in Predicting the Prognosis of Acute Cerebral Infarction with Endovascular Treatment

**DOI:** 10.2174/1573405619666230201103813

**Published:** 2023-05-31

**Authors:** Zhonghai Tao, Fang Zhou, Haojiang Zhang, Mingyue Qian

**Affiliations:** 1 Department of Neurology, Second People’s Hospital of Lianyungang, Lianyungang, Jiangsu, China;; 2 Department of Neurology, Lianyungang Cancer Hospital, Lianyungang, Jiangsu, China

**Keywords:** FVH, ASPECTS, endovascular treatment, acute cerebral infarction, mRS, FLAIR, mRS, FLAIR

## Abstract

**Objective:**

To explore the MRI T2 fluid-attenuated inversion recovery (FLAIR) vascular hyperintensities (FVH) combined with diffusion-weighted imaging (DWI) Alberta Stroke Program Early CT Score (ASPECTS) in predicting the prognosis of acute cerebral infarction (ACI) with endovascular treatment.

**Methods:**

The patients with ACI in the anterior circulation who underwent endovascular treatment from June 2016 to December 2020 were divided into a good prognosis group and a poor prognosis group according to the modified Rankin Scale (mRS) score at 90 days after the operation. The differences in general clinical baseline data, CT-ASPECTS, FVH, and DWI-ASPECTS between the two groups were analyzed. The receiver operating characteristic (ROC) curve was used to analyze the predictive power of prediction models on prognosis.

**Results:**

The results of the Binomial Logistic regression equation showed initial National Institute of Health stroke scale (NIHSS), Mori grade, DWI-ASPECTS, and FVH were independent risk factors for prognosis. The predictive power of the FVH + DWI-ASPECTS prediction model was highest, and the predictive power of DWI-ASPECTS was higher than that of CT-ASPECTS.

**Conclusion:**

DWI-ASPECTS is better than CT-ASPECTS in predicting the prognosis of ACI with endovascular treatment, and the combined prediction model of FVH and DWI-ASPECTS has higher prediction performance, which can be used as a preoperative evaluation method to predict the effect of endovascular treatment for ACI.

## 1 INTRODUCTION

The ultra-early treatment for acute cerebral infarction (ACI) is very important to the patient's prognosis [1]. The main treatment method is reperfusion therapy, which allows the occluded blood vessels to open again as soon as possible, restores blood flow, and saves the ischemic penumbra tissue. Reperfusion treatment methods include intravenous thrombolytic therapy and endovascular therapy. As one of the main methods of reperfusion therapy for ACI, endovascular therapy is increasingly being clinically applied [2, 3]. However, the evaluation of the safety and effectiveness of endovascular therapy should not be ignored in clinical work.

With the development of imaging technology, more and more studies have shown that some imaging indicators can predict the prognosis of ACI with endovascular treatment. Several CT and MRI parameters are considered to be risk indicators for patients with ACI after endovascular therapy [4, 5]. Alberta Stroke Program Early CT Score (ASPECTS) is a semi-quantitative scoring system for assessing early ischemic changes in the middle cerebral artery area. Its main purpose is to make treatment decision-making simple, fast, and reliable. The scoring system was originally designed for plain CT, with 10 points representing a normal brain, and 0 points representing diffuse ischemic changes in the middle cerebral artery area [6]. The scoring system was originally designed for plain CT and has been gradually applied to diffusion-weighted imaging (DWI) images [7-9], although there are literature reports their value in predicting the prognosis of ACI with endovascular treatment [5, 7], there are few reports about the comparative analysis of the two. MR angiography (MRA) is increasingly used to evaluate blood vessels because of its advantages in accurately displaying blood vessels without the need to inject a contrast agent [10]. MRA can accurately assess large intracranial vessel occlusion, but MRA is not as good as CTA in evaluating collateral compensation. MRI T2 fluid-attenuated inversion recovery (FLAIR) vascular hyperintensities (FVH) can find the occurrence of ACI earlier than DWI. MRA combined with FVH can make up for the shortcomings of MRA.

This study aimed to compare and analyze the predictive power of CT scan ASPECTS score and DWI ASPECTS score on prognosis, and to explore the value of FVH combined with DWI ASPECTS score in predicting the prognosis of ACI with endovascular treatment.

## 2 METHODS

### 2.1 Patients

Sixty-five patients with ACI in the anterior circulation who underwent endovascular treatment in the Department of Neurology of our hospitals from June 2016 to December 2020 were selected as the research objects. Inclusion criteria: ① Age ≥ 18 years old; ② within the 24 h interval between symptom onset and operation; ③ ASPECTS ≥ 6; ④ National Institute of Health stroke scale, (NIHSS) ≥ 6; ⑤ No operation contraindications.

This study was approved by the ethics committee of the Second People’s Hospital of Lianyungang (2015 x 033). Due to the retrospective nature of the study, informed consent was waived.

### 2.2 Groups

This study was a retrospective analysis of the data collected from the hospital. All patients were confirmed with anterior circulation great vessel occlusion by MRA. The endovascular treatment was performed according to the hospital’s protocol. Sixty-five patients were divided into a good prognosis group (modified Rankin Scale, [mRS] ≤ 2, 22 cases) and a poor prognosis group (mRS ≥ 3, 43 cases) according to the mRS at 90 days after the operation.

### 2.3 Clinical Data Collection

The clinical data analyzed in this study included: age, gender, high blood pressure (HBP) history, diabetes mellitus (DM) history, atrial fibrillation onset (AF) history, onset to reperfusion time, stroke classification (was assessed with Trial of ORG 10172 in Acute Stroke Treatment TOAST) [11], FVH, initial NIHSS, 24 h NIHSS after endovascular treatment, 90 d NIHSS after endovascular treatment, 90 d mRS after endovascular treatment, CT-ASPECTS, DWI-ASPECTS, vascular recanalization (was assessed with Mori classification) [12].

### 2.4 Image Analysis

Baseline CT image or DWI was used to grade the ASPECTS [9] standardized 10-point scale by two-stroke neurologists. Two raters achieved one common CT-ASPECTS or DWI- ASPECTS per patient through consensus during a joint reading session.

SIMENS spectra HD x 3.0T MR scanner with an 8-channel cranial phased array coil was used for detection. The scan range was from the lower end of the medulla oblongata to the top of the skull. T2-FLAIR sequence: TR 85 00.0 ms, TE 145.0 ms, FOV 240 mm × 240 mm, matrix 320 × 224, NAX 1, layer thickness 6 mm, spacing 1 mm.

The Olindo scoring method was used to evaluate the FVH [13]. Scoring criteria: the initial appearance of the middle cerebral artery M1 segment as the first layer was selected, 10 layers from bottom to top were observed, if there was no layer showing FVH, it was recorded as 0 points, if there was a layer of FVH positive, 1 point was added, the maximum was 10 points.

**Fig. (1):**
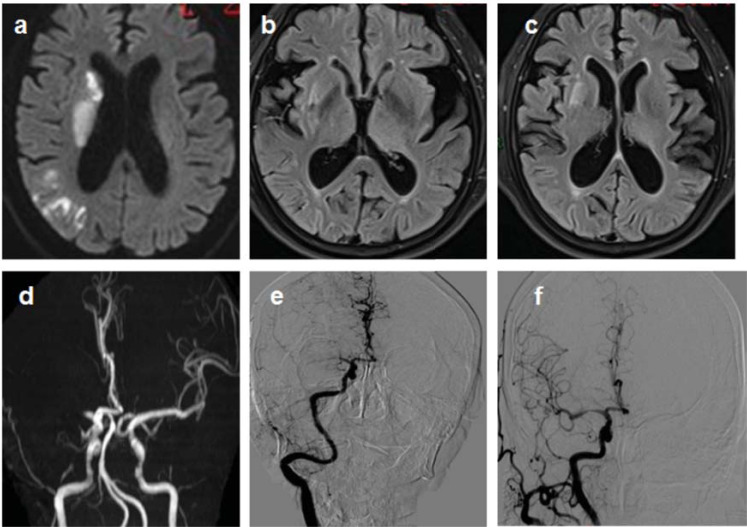
A 67-year-old female patient was admitted to the hospital because she was “discovered with weakness in her left limb for 3.5 hours”. (**a**) DW image showed the infarct of the right middle cerebral artery blood supply area. (**b** and **c**) FVH in the blood supply area of the middle cerebral artery. (**d**) MRA: right middle cerebral artery occlusion. (**e**) DSA: right middle cerebral artery occlusion. (**f**) The right middle cerebral artery was successfully opened 196min after admission.

A 67-year-old female patient was admitted to the hospital because she was “discovered with weakness in her left limb for 3.5 hours”. MRA indicated occlusion of the right middle cerebral artery. The CT-ASPECT score was 7 points, and the DWI-ASPECT score was 5 points, suggesting that the DWI-ASPECT score could better reflect the situation of the infarct. The FVH score was 5 points, which indicated the existence of collateral circulation. After intra-arterial thrombus removal, the patient’s NIHSS score dropped from 23 to 11 points (Fig. **1**).

### 2.5 Statistical Analysis

Statistical analysis was performed using SPSS22.0 software. Enumeration data was represented by composition ratio, and the chi-square test was used for comparison between groups. Normally distributed measurement data were represented by mean ± SD, and comparison between groups was performed by independent t-tests. The measurement data of skewed distribution were represented by median and range, and the Mann-Whitney U test was used for comparison between groups. Binomial Logistic regression analysis was used to analyze independent risk factors for prognosis. The combined prediction model of FVH and DWI-ASPECTS was constructed with Logistic regression. The receiver operating characteristic (ROC) curve was used to analyze the predictive power on prognosis, and the area under the ROC curve (AUC) was compared with the DeLong test. *p* < 0.05 was considered statistically significant.

## 3 RESULTS

### 3.1 Comparison of Clinical Baseline Data Between the Two Groups

The age, CT-ASPECTS, DWI- ASPECTS of the good prognosis group were higher than that of the poor prognosis group, the NIHSS of the good prognosis group was lower than that of the poor prognosis group, the FVH score ofthe good prognosis group was higher than that of poor prognosis group, and the differences between groups were statistically significant (*p* < 0.05). There was no statistically significant difference in other baseline data between groups (*p* > 0.05). The results were shown in (Table **1**).

### 3.2 Independent Risk Factors for Prognosis

Substituting factors such as age, Initial NIHSS or CT-ASPECTS, DWI- ASPECTS, Mori grade, and FVH were taken into the Binomial Logistic regression equation. The results of the Binomial Logistic regression equation showed initial NIHSS (OR = 0.886, 95%CI: 0.815-0.964), Mori grade (OR = 0.054, 95%CI: 0.005-0.621), DWI- ASPECTS (OR = 4.049, 95%CI: 1.429-11.476), FVH (OR = 0.017, 95%CI: 0.001-0.385) were independent risk factors for prognosis. The results are shown in (Table **2**).

### 3.3 The Predictive Power of CT-ASPECTS, DWI-ASPECTS and FVH + DWI-ASPECTS Prediction Model on Prognosis

**Fig. (2):**
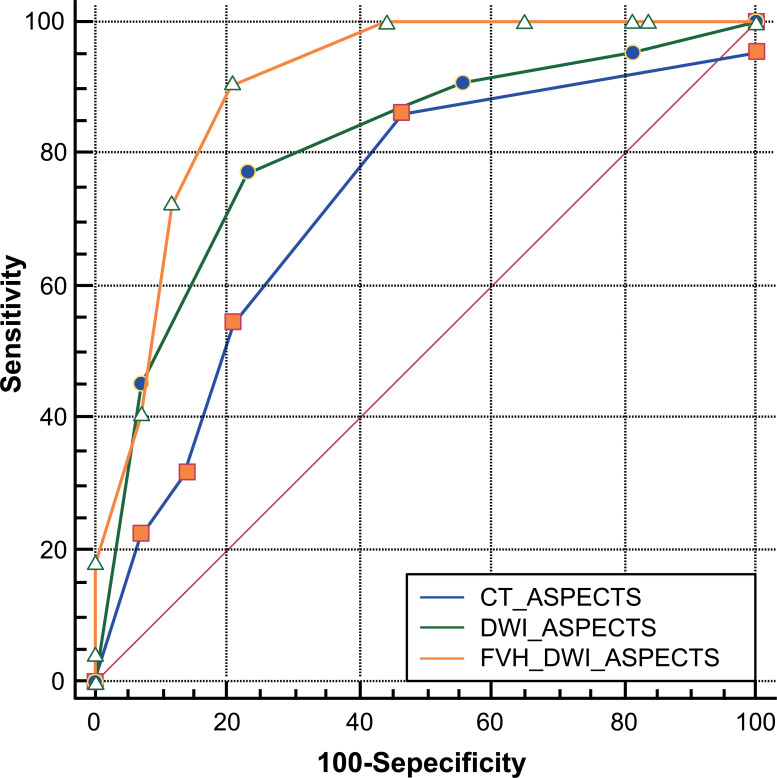
Predictive power of CT-ASPECTS, DWI-ASPECTS and FVH + DWI-ASPECTS prediction model on prognosis of endovascular treatment for ACI were analyzed with ROC curve.

The ROC curve was used to analyze the predictive power of CT-ASPECTS, DWI-ASPECTS, and FVH + DWI-ASPECTS prediction model on prognosis. The results showed the area under curve (AUC) of CT-ASPECTS, DWI-ASPECTS and FVH + DWI-ASPECTS prediction model on prognosis were 0.724 (95%CI: 0.599-0.82), 0.810 (95%CI: 0.693-0.896), 0.915 (0.820-0.970) respectively. The predictive power of the FVH + DWI-ASPECTS prediction model was highest, and the predictive power of DWI-ASPECTS was higher than that of CT-ASPECTS, and the differences between groups were statistically significant (*p* < 0.05) (Table **3** and Fig. **2**).

## 4 DISCUSSION

Cerebral infarction refers to the blood supply disorder of the brain caused by various reasons, leading to ischemia and hypoxic necrosis of brain tissue. It is a common disease that seriously endangers human health. Cerebral infarction and cerebral hemorrhage are collectively referred to as a stroke, the fatality rate ranks second among all diseases in the world, and it is the primary cause of disability [14]. Endovascular therapy can significantly improve the prognosis of ACI, but many factors can significantly affect the effect of endovascular therapy. Exploring the factors that affect the efficacy of endovascular therapy, and finding simple and effective methods to predict the efficacy of endovascular therapy has always been a hot spot in clinical research.

This study firstly analyzed the clinical baseline data of the two groups, the results showed patients in the good prognosis group were younger and had a better neurological function, collateral circulation, and ASPECTS on admission, and vascular recanalization was better, which was basically consistent with the other researches [15-17]. Then, the Binomial Logistic regression analysis was used to analyze the independent risk factors for prognosis, the results showed that initial NIHSS, Mori grade, DWI- ASPECTS, and FVH score were the independent risk factors for prognosis. At last, The ROC curve was used to analyze the predictive power of CT-ASPECTS, DWI-ASPECTS, and FVH + DWI-ASPECTS prediction model on prognosis, the results showed the AUC of FVH + DWI-ASPECTS prediction model was highest, and the AUC of DWI-ASPECTS was higher than that of CT-ASPECTS. Our results indicated that the predictive power of DWI-ASPECTS was better than that of CT-ASPECTS, which may be related to the limitations of CT for the early detection of acute infarction lesions, while DWI can accurately assess the condition of infarcts early and is more conducive to the judgment of the disease [8, 9]. MRA can accurately display the blockage of large blood vessels and the FVH score displays the compensation by the front and back communicating arteries. Collateral circulation compensation is a very important factor, which affects the volume of the core infarct area and the size of the ischemic penumbra. CTA is superior to MRA in assessing collateral circulation but inferior to MRI in judging infarct focus [18-20]. MRA combined with FVH can make up for the lack of MRA in the evaluation of collateral circulation. The application of MRI can evaluate the condition of the infarct focus and the condition of the blood vessel at the same time, which is more conducive to the correct selection of patients for endovascular treatment. Therefore, we used Logistic regression to construct the combined prediction model of FVH and DWI-ASPECTS in predicting the prognosis of ACI with endovascular treatment and got good predictive power. However, the number of samples in this study is small, and the above results need to be confirmed by further research.

## CONCLUSION

In summary, DWI-ASPECTS is better than CT-ASPECTS in predicting the prognosis of ACI with endovascular treatment, and the combined prediction model of FVH and DWI-ASPECTS has higher prediction performance, which may be used as a preoperative evaluation method to predict the effect of endovascular treatment for ACI.

## Figures and Tables

**Table 1:** Comparison of clinical baseline data between the two groups.

**-**	**Good Prognosis Group (n = 22)**	**Poor Prognosis Group (n = 43)**	***p***
Age	64.91 ± 9.73	70.88 ± 11.68	0.04
Gender (Male/Female)	16/6 (72.73% / 27.27%)	26/17 (60.47% / 39.53%)	0.33
HBP (Y/N)	16/6 (72.73%/ 27.27%)	28/15 (65.12% / 34.88%)	0.54
DM (Y/N)	12/10 (54.55% / 45.45%)	26/17 (60.47% / 39.53%)	0.65
AF (Y/N)	3/19 (13.64% / 86.36%)	8/35 (18.60% / 81.40%)	0.61
Onset to reperfusion time (min)	517 (179-1250)	586 (258-1440)	0.34
CT-ASPECTS	8 (4-10)	6 (6-10)	<0.01
DWI- ASPECTS	9 (6-10)	8 (6-10)	<0.01
Initial NIHSS	11 (6-31)	19 (6-34)	<0.01
24 h NIHSS after endovascular treatment	3.5 (0-9)	14 (4-30)	<0.01
90 d NIHSS after endovascular treatment	2 (0-3)	10 (2-20)	<0.01
Mori grade	-	-	0.01
1	1 (4.55%)	1 (2.33%)	-
2	1 (4.55%)	8 (18.60%)	-
3	9 (40.91%)	29 (67.44%)	-
4	11 (50.00%)	5 (11.63%)	-
FVH score	6 (4-9)	3 (0-7)	< 0.01
TOAST classification	-	-	0.67
Large-artery atherosclerosis	12 (54.55%)	23 (53.49%)	-
Mortality (Y/N)	0/22 (0.00% / 100.00%)	2/41 (4.65% / 95.35%)	0.55

**Abbreviations:** AF: atrial fibrillationonset; ASPECTS: Alberta Stroke Program Early CT Score; DM: diabetes mellitus; HBP: high blood pressure; FVH: fluid-attenuated inversion recovery (FLAIR) vascular hyperintensities (FVH); NIHSS: National Institute of Health stroke scale; TOAST: Trial of ORG 10172 in Acute Stroke Treatment.

**Table 2:** Independent risk factors for prognosis.

**-**	**B**	**S.E,**	**Wald**	***P***	**OR**	**95%CI**
DWI- ASPECTS	1.399	0.532	6.922	0.009	4.049	1.429-11.476
FVH score	-4.103	1.606	6.529	0.011	0.017	0.001-0.385
Mori grade	-2.917	1.246	5.485	0.019	0.054	0.005-0.621
Initial NIHSS	-0.121	0.043	7.898	0.005	0.886	0.815-0.964

**Abbreviations:** ASPECTS: Alberta Stroke Program Early CT Score; FVH: fluid-attenuated inversion recovery (FLAIR) vascular hyperintensities (FVH); NIHSS: National Institute of Health stroke scale.

**Table 3:** Predictive power of prediction model on prognosis.

**-**	**Cutoff Value**	**AUC (95% CI)**	**Sensitivity (%)**	**Specificity (%)**	**Accuracy (%)**
CT-ASPECTS	6	0.724(0.599-0.827)	19/22 (86.36)	23/43 (53.49)	42/65 (64.62)
DWI-ASPECTS	8	0.810(0.693-0.896)*	17/22 (77.27)	33/43 (76.74)	50/65 (76.92)
FVH+DWI-ASPECTS	0.232	0.915(0.820-0.970)*#	19/22 (86.36)	35/43 (81.40)	54/65 (83.08)

**Abbreviations:** ASPECTS: Alberta Stroke Program Early CT Score; FVH: fluid-attenuated inversion recovery (FLAIR) vascular hyperintensities (FVH); MRA: MR angiography. *vs. CT-ASPECTS, *p* < 0.05; # *vs*. DWI-ASPECTS, *p* < 0.05.

## Data Availability

The data that support the findings of this study are available from the corresponding author [Z.T], upon reasonable request.

## LIST OF ABBREVIATIONS

DWI: Diffusion-Weighted Imaging
FLAIR: Fluid-Attenuated Inversion Recovery
mRS: Modified Rankin Scale
